# DOCK3 orchestrates metastasis and immune microenvironment in prostate cancer

**DOI:** 10.3389/fruro.2025.1662692

**Published:** 2025-10-22

**Authors:** Jiaxue Han, Ming Zhang, Haipeng Zhou, Qiao Xiong, Xin Zhong, Ping Tan

**Affiliations:** Department of Urology, West China Hospital of Sichuan University, Chengdu, China

**Keywords:** DOCK3, metastasis, prostate cancer, immune microenvironment, CDKN3

## Abstract

**Introduction:**

Prostate cancer (PCa) is a leading cause of male cancer mortality, with metastasis and immune evasion posing major therapeutic challenges. DOCK3, a guanine nucleotide exchange factor implicated in cytoskeletal dynamics, is poorly characterized in PCa. This study investigates DOCK3’s role in PCa metastasis and tumor immune microenvironment (TIME) remodeling.

**Methods:**

Multi-omics analyses integrated bulk RNA-seq from TCGA-PRAD (499 tumors/52 normals), scRNA-seq from GEO (45,325 cells), and genomic data. We performed: Differential expression analysis (DESeq2), Immune deconvolution (CIBERSORT,ssGSEA, xCell), WGCNA co-expression networks, Tumor mutational burden (TMB) assessment, Distant metastasis (M1 vs. M0) association studies, scRNA-seq clustering (Harmony/UMAP) and DE testing. Statistical significance thresholds: |log2FC|>1, padj<0.05.

**Results:**

DOCK3 expression was found to be significantly elevated in metastatic (M1) tumors compared to primary (M0) tumors (p<0.05) and demonstrated a strong positive correlation with a higher tumor mutational burden (TMB) in metastatic samples (p<0.001). Cellular specificity analysis revealed that DOCK3 was exclusively and highly enriched within malignant epithelial and stromal cells, specifically in Cluster 6, where it exhibited a log2 fold-change of 9.13 (padj<1e-200) and was expressed in 54% of cells, compared to a negligible presence in all other clusters. In the tumor microenvironment, elevated DOCK3 expression was associated with a significant increase in cytotoxic immune infiltration, notably of CD8^+^ T and Natural Killer cells, a finding consistently supported by multiple computational algorithms (all p<0.05). Clinically, a high level of DOCK3 was significantly associated with metastatic status (p<0.01), whereas high expression of CDKN3 was correlated with advanced disease features, including higher Gleason scores (3-5) and T-stage (T2-T4) (p<0.01). Furthermore, significant differences in immune infiltration patterns were observed between clusters. Pathway enrichment analysis of genes co-expressed with DOCK3, identified through the WGCNA Green Module, indicated significant involvement in biological processes such as cytoskeletal reorganization, muscle contraction, and metabolic pathways (FDR<0.01).

**Conclusion:**

DOCK3 drives PCa metastasis through cytoskeletal dynamics while paradoxically promoting an immunologically active microenvironment. Its tumor-specific expression and association with aggressive clinical features nominate DOCK3 as a novel biomarker for risk stratification and a promising therapeutic target for combinatorial immunotherapy in immunologically “cold” PCa.

## Introduction

Prostate cancer (PCa) is the second most common cancer among men worldwide and the fifth leading cause of cancer-related deaths (1). There are nearly 1.4 million new cases of PCa and approximately 375,000 deaths annually worldwide (2). Despite significant advances in understanding the molecular mechanisms of PCa, the specific molecular pathways involved in promoting metastasis and immune evasion remain unclear (3, 4). Recent studies have highlighted the importance of several key genes and molecular pathways, including Dedicator of Cytokinesis 3 (DOCK3), in tumor progression and therapeutic response, drawing attention to the potential role of DOCK3 in various cancers (5, 6).

DOCK3 is a member of the DOCK family of guanine nucleotide exchange factors (GEFs), which interacts with other cancer-related signaling pathways, such as the actin cytoskeleton and focal adhesion pathways, playing a critical role in regulating cytoskeletal dynamics (7, 8). It is involved in multiple cellular processes, including migration, adhesion, and invasion, suggesting its potential role as a key regulator of cancer metastasis. Previous studies have shown that DOCK3 is associated with the development of colorectal cancer and serves as an independent prognostic risk factor for colorectal adenocarcinoma, making it a promising biomarker for prognosis prediction in colorectal cancer (9, 10). In small cell lung cancer, DOCK3 mediates tumor cell adhesion, migration, and invasion through activating the RAC1 signaling pathway (11). However, the role of DOCK3 in PCa metastasis and its regulatory mechanisms in the tumor immune microenvironment remain to be further elucidated.

This study aims to systematically investigate the expression characteristics of DOCK3 in PCa by integrating multi-omics data, including bulk RNA sequencing, single-cell RNA sequencing, and genomic data. The goal is to elucidate the potential role of DOCK3 in the metastatic progression of PCa and its modulation of immune responses, thereby establishing its significance as a target for immunotherapy. Additionally, we will evaluate the correlation between DOCK3 expression levels and clinical features such as Gleason score and tumor stage, providing theoretical support for the development of DOCK3-based prognostic biomarkers.

## Methods

### Bulk RNA-seq analysis methods

Data Acquisition and Preprocessing: Transcriptomic data and clinical information for prostate adenocarcinoma (PRAD) were retrieved from The Cancer Genome Atlas (TCGA) database using the TCGAbiolinks R package(version 2.36.0). The dataset included gene expression matrices (raw counts and transcripts per million (TPM) from tumor samples (primary solid tumors, 01A) and adjacent normal tissue samples (11A). Gene expression data (TPM + 1) were log2-transformed. Duplicate samples and low-expression genes were subsequently removed.

#### Differential expression analysis

Differential expression analysis between tumor and normal samples was performed using the DESeq2 package(version 1.48.1). Genes with an absolute log2FoldChange >1 and an adjusted p-value (padj)<0.05 were defined as significantly differentially expressed. Results were visualized via heatmap and volcano plot.

#### Immune infiltration analysis

The ESTIMATE algorithm(via the estimate package (v1.0.13)) was employed to calculate immune scores, stromal scores, and tumor purity for tumor samples. The relative abundance of 22 immune cell types was inferred using the CIBERSORT (via the e1071 package(v1.7-14) and preprocessCore package(v1.70.0)), ssGSEA (via the fgsea package (v1.34.0)), and xCell(via the xCell package (v1.1.0)) algorithms. Differences in immune infiltration between groups with high and low DOCK3 expression were compared.

#### Tumor mutational burden analysis

The TMB landscape of prostate cancer was systematically analyzed. An overview plot was generated to display mutation frequency, distribution of variant types, frequently mutated genes, and sample-level mutational load. A boxplot visualized the mutation frequency of the top 50 mutated genes. The Wilcoxon rank-sum test was applied to compare TMB values between clinical subgroups (e.g., metastatic status M0 vs. M1) for statistical significance.

#### Gene co-expression network analysis

A weighted gene co-expression network analysis (WGCNA) was executed. A soft thresholding power(b=2) was selected to construct an adjacency matrix based on a topological overlap matrix (TOM). Modules were identified using the dynamic tree cut method. Module-trait relationships were assessed by correlating module eigengenes with immune scores. Genes within the green module, which contained DOCK3, were subjected to Gene Ontology (GO) and Kyoto Encyclopedia of Genes and Genomes (KEGG) pathway enrichment analyses.

#### Distant metastasis-associated differential analysis

Differential expression analysis was performed between samples with and without distant metastasis (M1 vs. M0), applying the same significance thresholds (|log2FC| >1, padj <0.05). Results were presented using a heatmap and a volcano plot.

#### DOCK3-specific in-depth analysis

The intersection of distant metastasis-associated differentially expressed genes and the top 50 TMB genes was taken, with a focused analysis on DOCK3. Gene Set Enrichment Analysis (GSEA)was conducted to evaluate pathway enrichment in groups with high and low DOCK3 expression.

#### Clustering analysis

Based on the expression differences of DOCK3 and CDKN3, tumor samples were categorized using the ConsensusClusterPlus algorithm(via the ConsensusClusterPlus package (v1.72.0)). Differences between clusters in terms of immune infiltration, gene expression profiles, and clinical characteristics were analyzed.

#### External validation

The differential expression of DOCK3 between tumor and normal tissues was validated in the GSE55945 dataset (comprising 13 tumor and 8 normal samples).

### Single-cell RNA-seq analysis methods

#### Data loading and quality control

10X Genomics single-cell RNA sequencing data from 9 prostate tissue samples (3 normal, 3 high-grade prostatic intraepithelial neoplasia (HGPIN), 3 tumor) was loaded. Quality control metrics included calculating the percentage of mitochondrial genes (mt_percent) and hemoglobin genes (HB_percent). Cells were retained if: nFeature_RNA >300 & <7000, mt_percent <10, HB_percent <3, and nCount_RNA >1000 & <97th percentile.

#### Data integration and batch correction

Data integration and batch effect correction were performed using the Harmony algorithm (key function: IntegrateLayers()), followed by dimensionality reduction steps: Performing principal component analysis (PCA) for initial dimensionality reduction, then applying Harmony for batch correction or data integration, and finally using UMAP or t-SNE for nonlinear visualization of the data in two or three dimensions.

#### Cell clustering

Cell clustering was explored at multiple resolutions. A resolution of 0.2 (modularity = 0.912) was selected. Doublets were identified and removed using DoubletFinder(via the DoubletFinder package (v2.0.6)).

#### Cell type annotation

Cell clusters were annotated using a three-pronged approach: 1) Automatic annotation with SingleR referencing the HumanPrimaryCellAtlas database; 2) Annotation using scCATCH based on prostate cancer-specific marker genes; 3) Manual annotation combining literature evidence and marker gene expression.

#### DOCK3-specific expression analysis

Differential expression analysis of DOCK3 across all cell clusters was performed to identify clusters with significant and specific DOCK3 expression.

## Results

**Figure 1:**
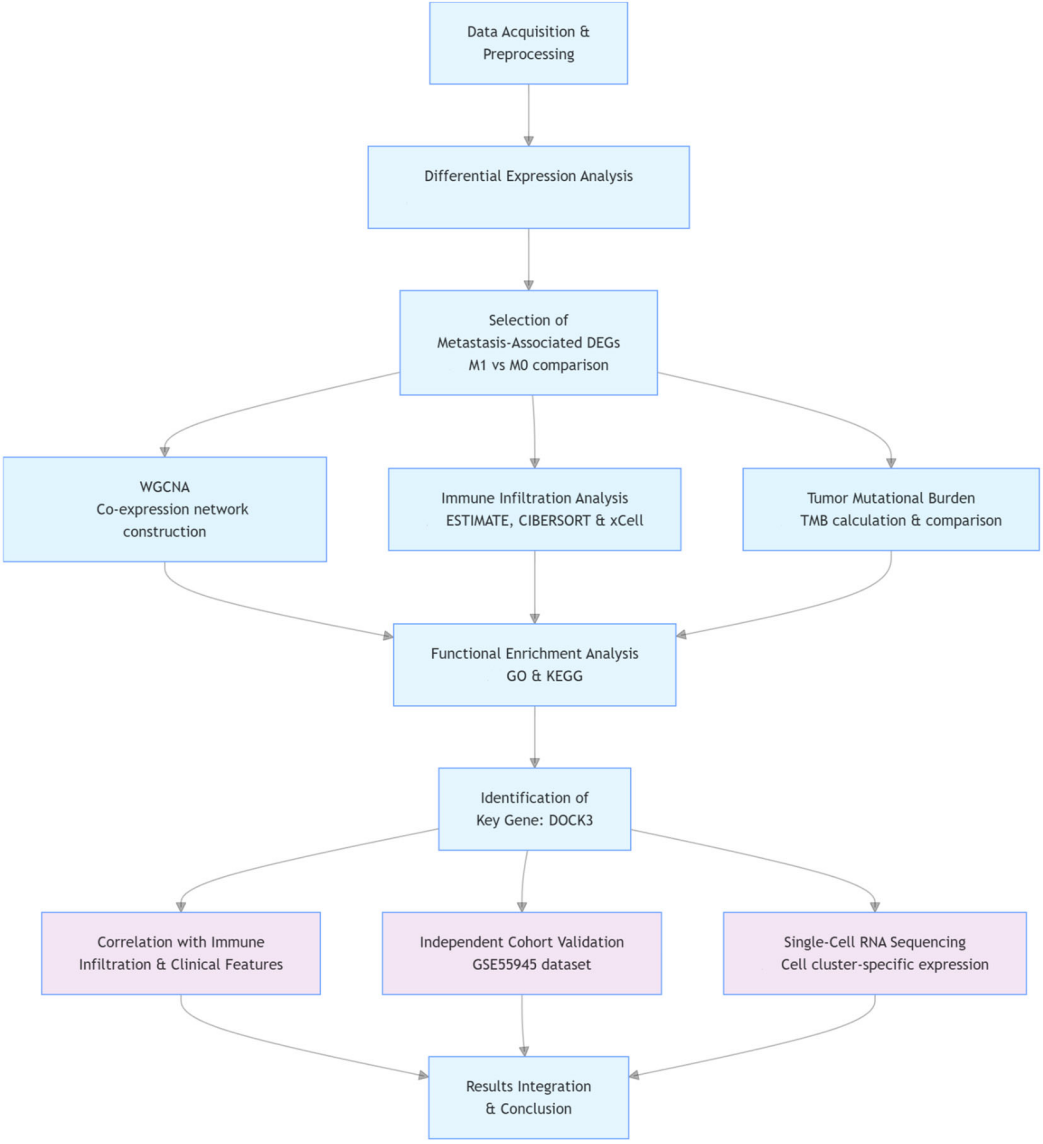
Schematic workflow of the integrated multi-omics analysis in this study.

The overall analytical workflow of this study is summarized in **Figure 1**.

### Bulk RNA-seq analysis results

#### Differential expression analysis

To identify transcriptomic alterations between prostate tumor and normal tissues, we performed differential gene expression analysis. The results successfully identified a set of differentially expressed genes (DEGs), including DOCK3 and CDKN3.

#### Distant metastasis-associated DEG analysis

**Figure 2:**
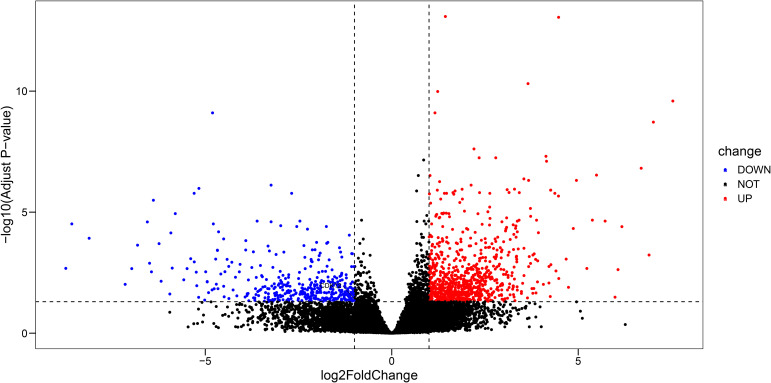
Volcano plot displaying differentially expressed genes (DEGs) between prostate cancer samples with distant metastasis (M1) and those without (M0) (|log2FC| > 1, adj. p < 0.05).

To uncover gene expression changes linked to metastatic progression, we identified DEGs between M1 and M0 groups. Notably, DOCK3 was identified as significantly differentially expressed genes in this comparison. The identified DEGs were visualized using a volcano plot (**Figure 2**).

#### Immune infiltration analysis

**Figure 3:**
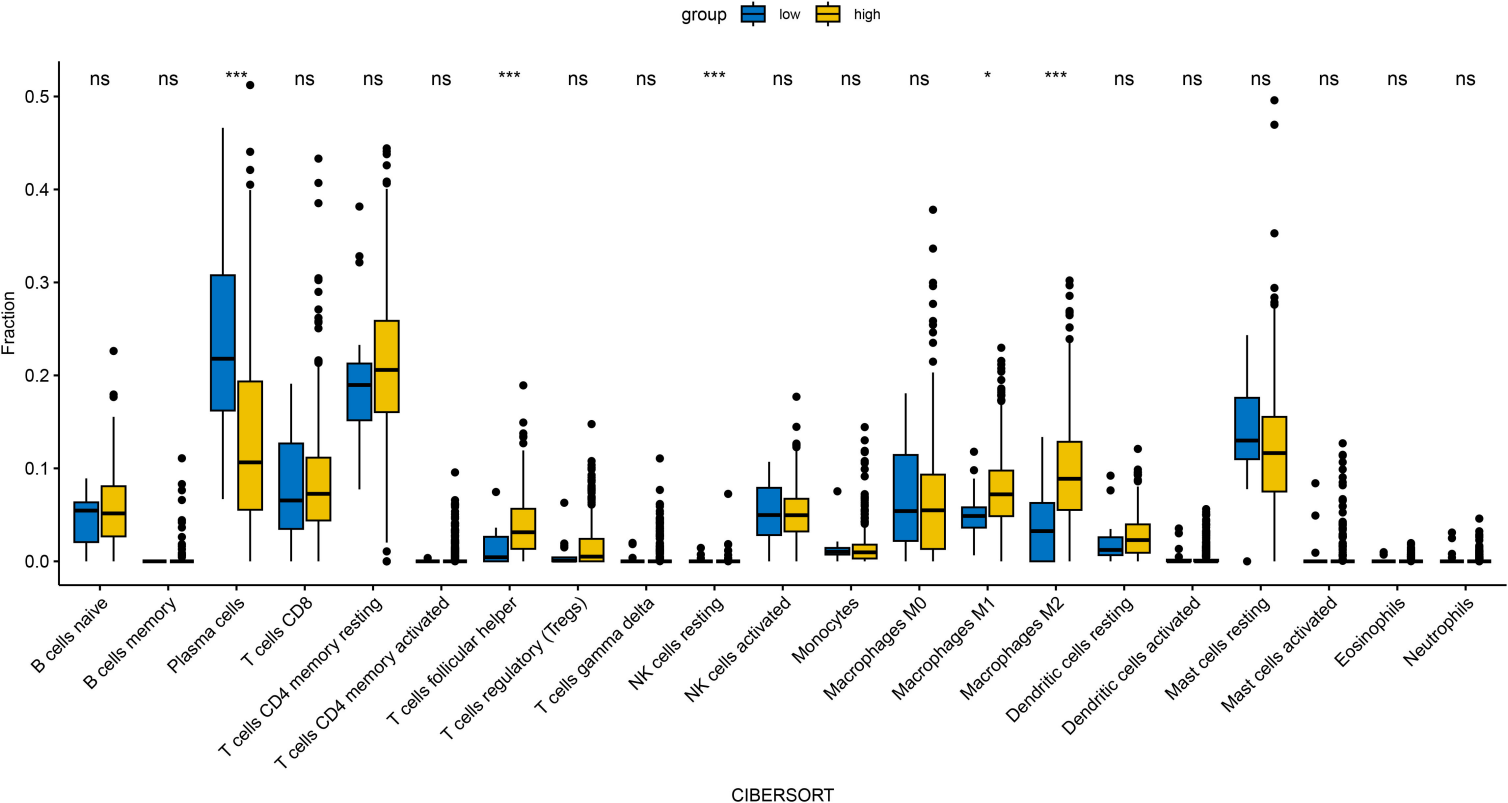
Comparative analysis of tumor immune infiltration landscapes between DOCK3-high and DOCK3-low expression groups, as assessed by CIBERSORT. *p < 0.05, ***p < 0.001.

**Figure 4:**
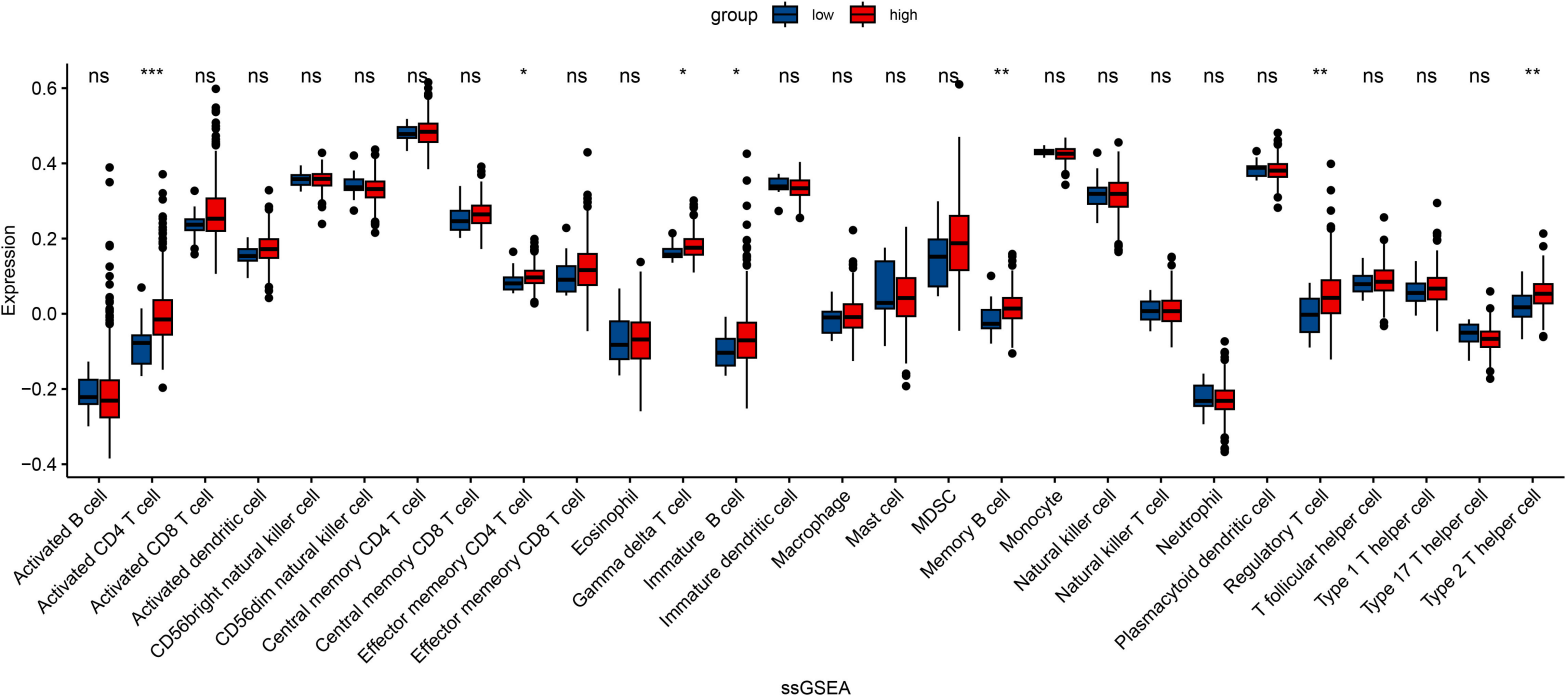
Single-sample Gene Set Enrichment Analysis (ssGSEA) showcasing the enrichment of immune-related pathways in samples stratified by DOCK3 expression. *p < 0.05, **p < 0.01, ***p < 0.001.

**Figure 5:**
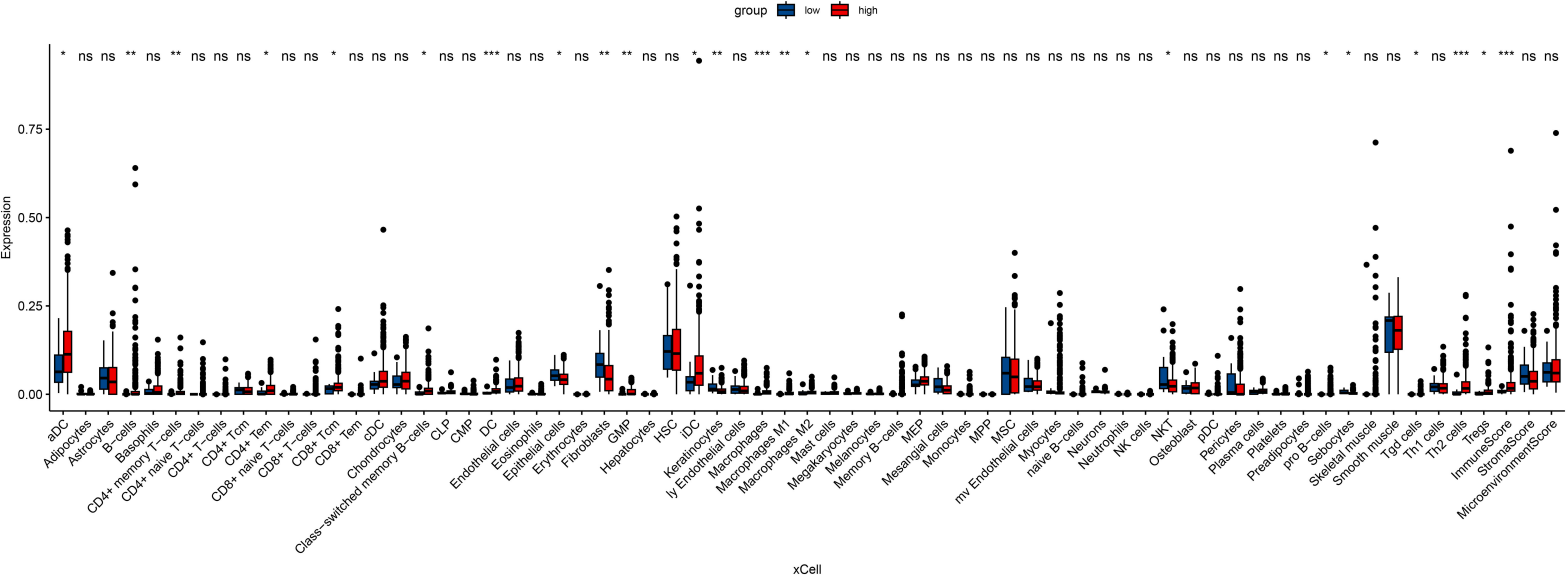
Evaluation of immune and stromal cell enrichment scores in DOCK3-high and DOCK3-low groups using the xCell algorithm. *p < 0.05, **p < 0.01, ***p < 0.001.

In an attempt to explore the tumor immune microenvironment, we estimated immune and stromal scores, tumor purity, and the relative abundance of 22 immune cell subtypes. Consistently across CIBERSORT (**Figure 3**), ssGSEA (**Figure 4**), and xCell (**Figure 5**) algorithms, samples with high DOCK3 expression exhibited significantly stronger immune cell infiltration (all p < 0.05).

#### Tumor mutational burden analysis

**Figure 6:**
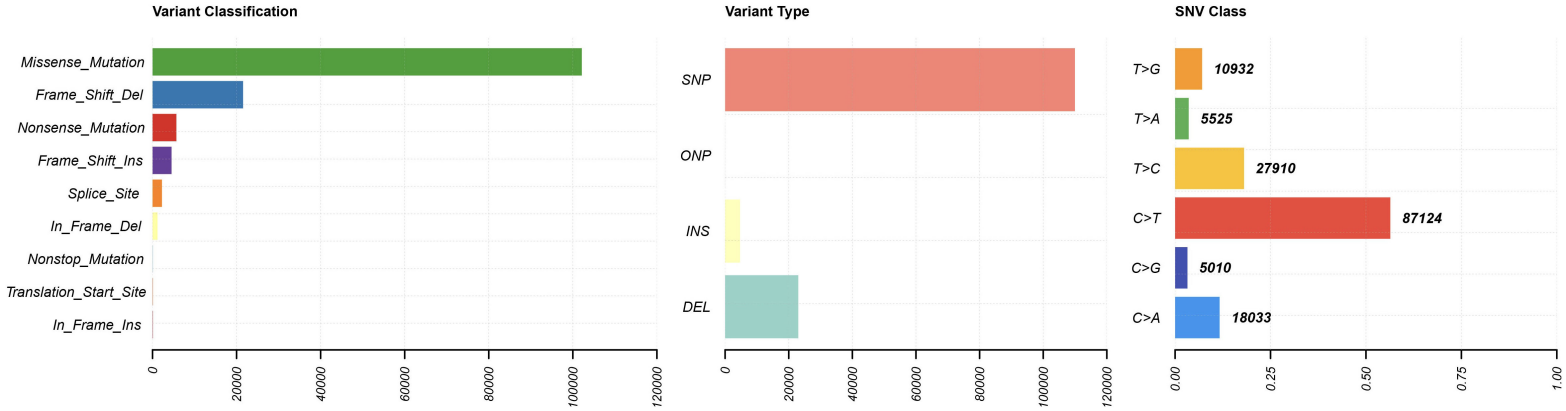
Tumor Mutational Burden (TMB) landscape.

To evaluate the genomic instability landscape of PRAD, we assessed tumor mutational burden (**Figure 6**). The results revealed that TMB was significantly higher in metastatic (M1) compared to non-metastatic (M0) samples (p < 0.001).

#### WGCNA

**Figure 7:**
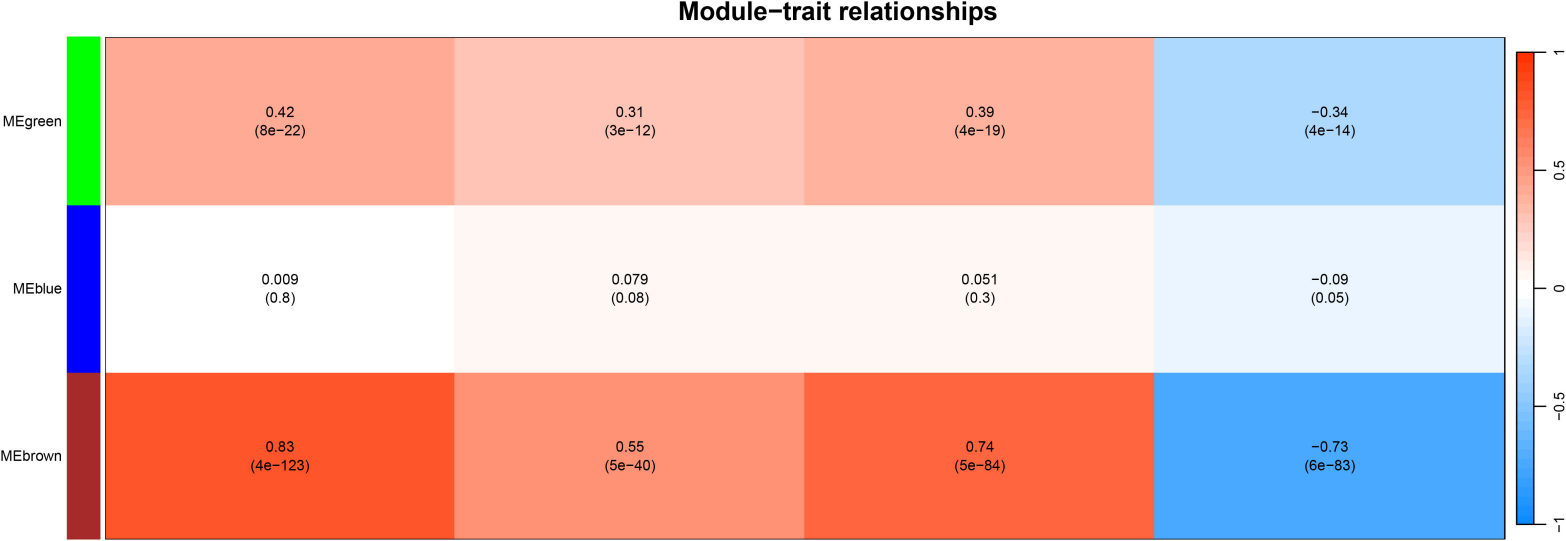
Weighted Gene Co-expression Network Analysis (WGCNA).

To identify co-expressed gene modules and their functional implications, we constructed a weighted gene co-expression network (**Figure 7**). The green module, which contains DOCK3, was significantly enriched in biological processes related to muscle contraction, cytoskeleton organization, and metabolic regulation (FDR < 0.01).

#### DOCK3-specific analysis

Given the potential multifunctional role of DOCK3, we examined its overlap with metastasis-associated DEGs and frequently mutated genes. We found that high DOCK3 expression was strongly associated with activation of immune-related pathways.

#### Clustering based on DOCK3 and CDKN3 expression

**Figure 8:**
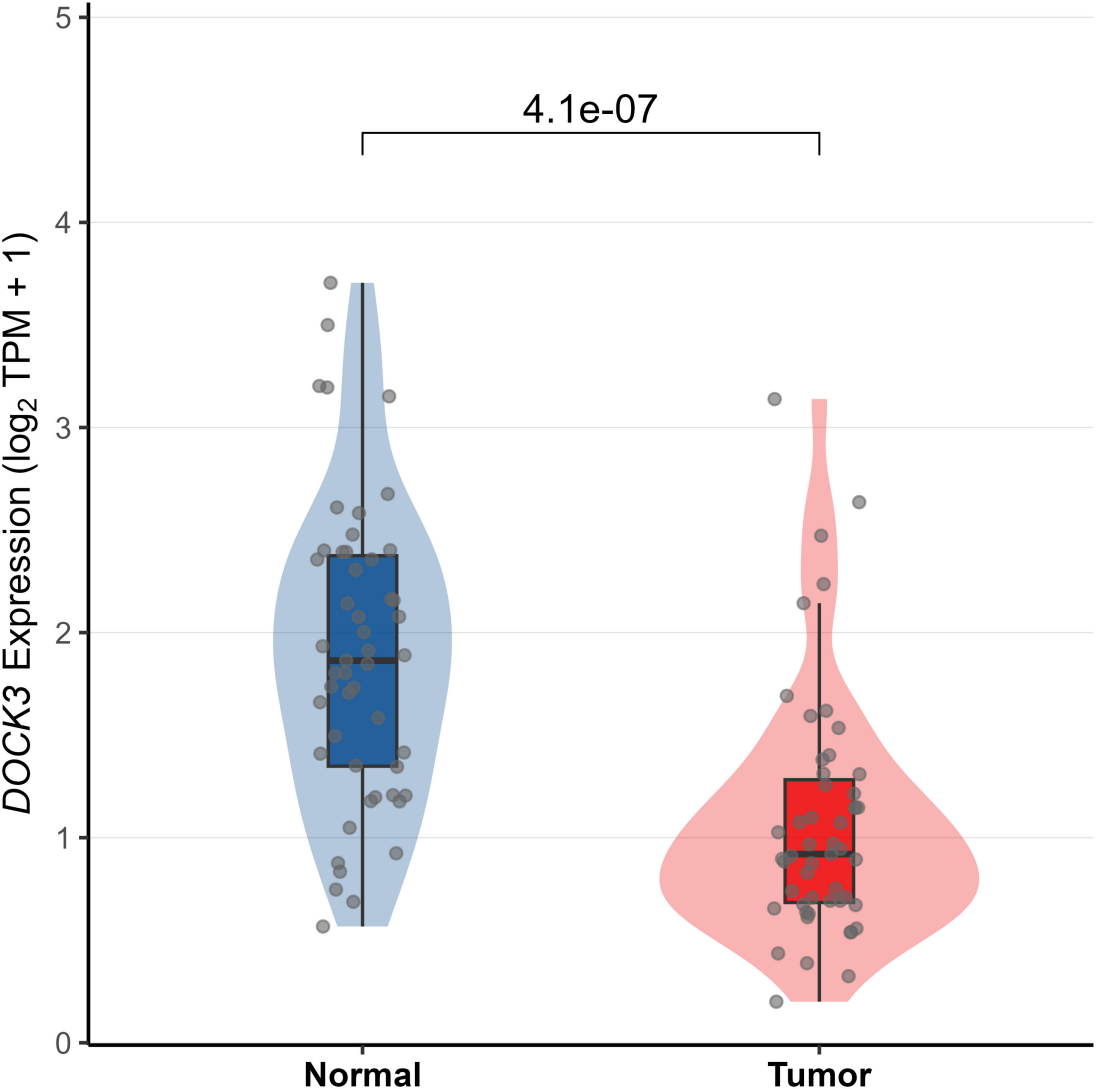
Differential expression of DOCK3 between prostate tumor tissues and normal adjacent tissues in the TCGA-PRAD cohort (|log2FC| > 1, adj. p < 0.05).

**Figure 9:**
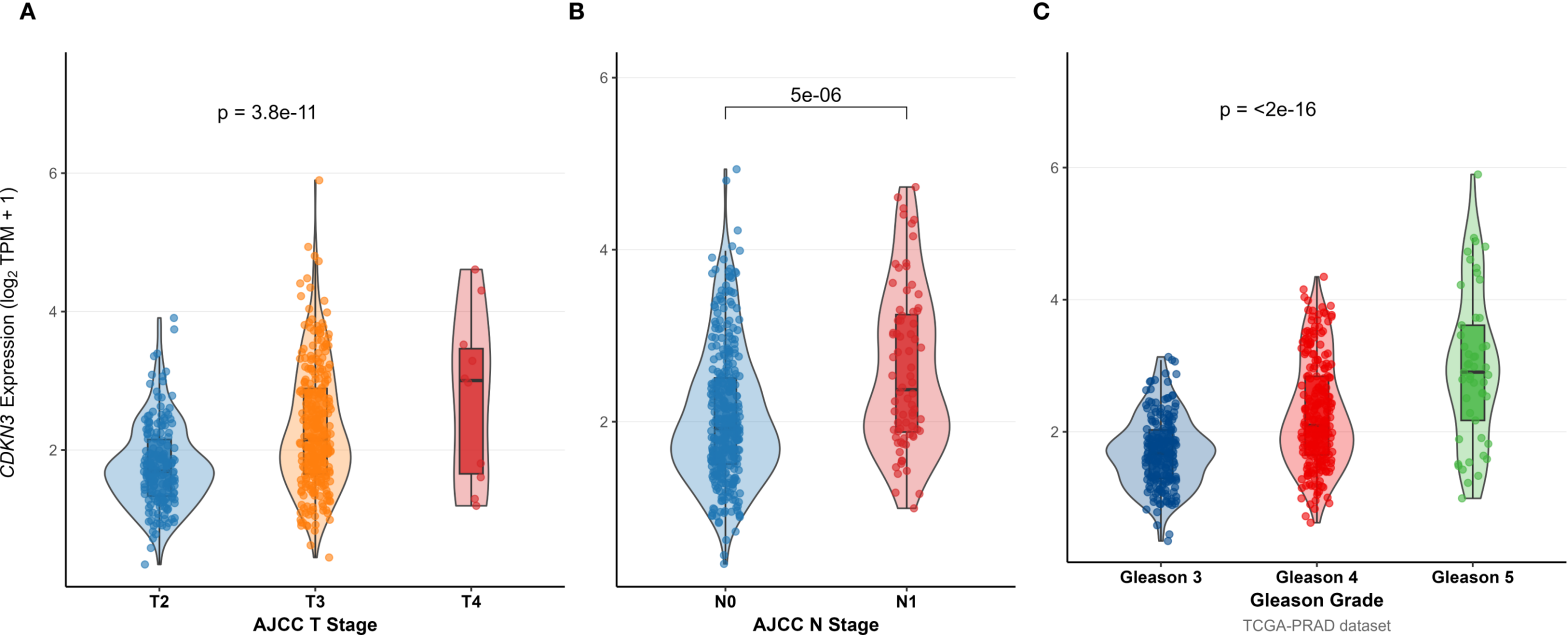
Association between CDKN3 expression levels and key clinical pathological features: **(A)** T stage, **(B)** N stage, and **(C)** Gleason Score (Grading).

**Figure 10:**
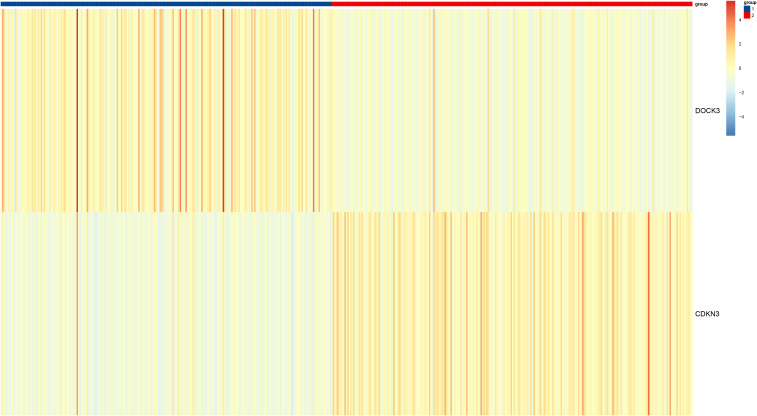
Heatmap of unsupervised clustering of tumor samples based on DOCK3 and CDKN3 expression patterns, alongside selected clinical features.

**Figure 11:**
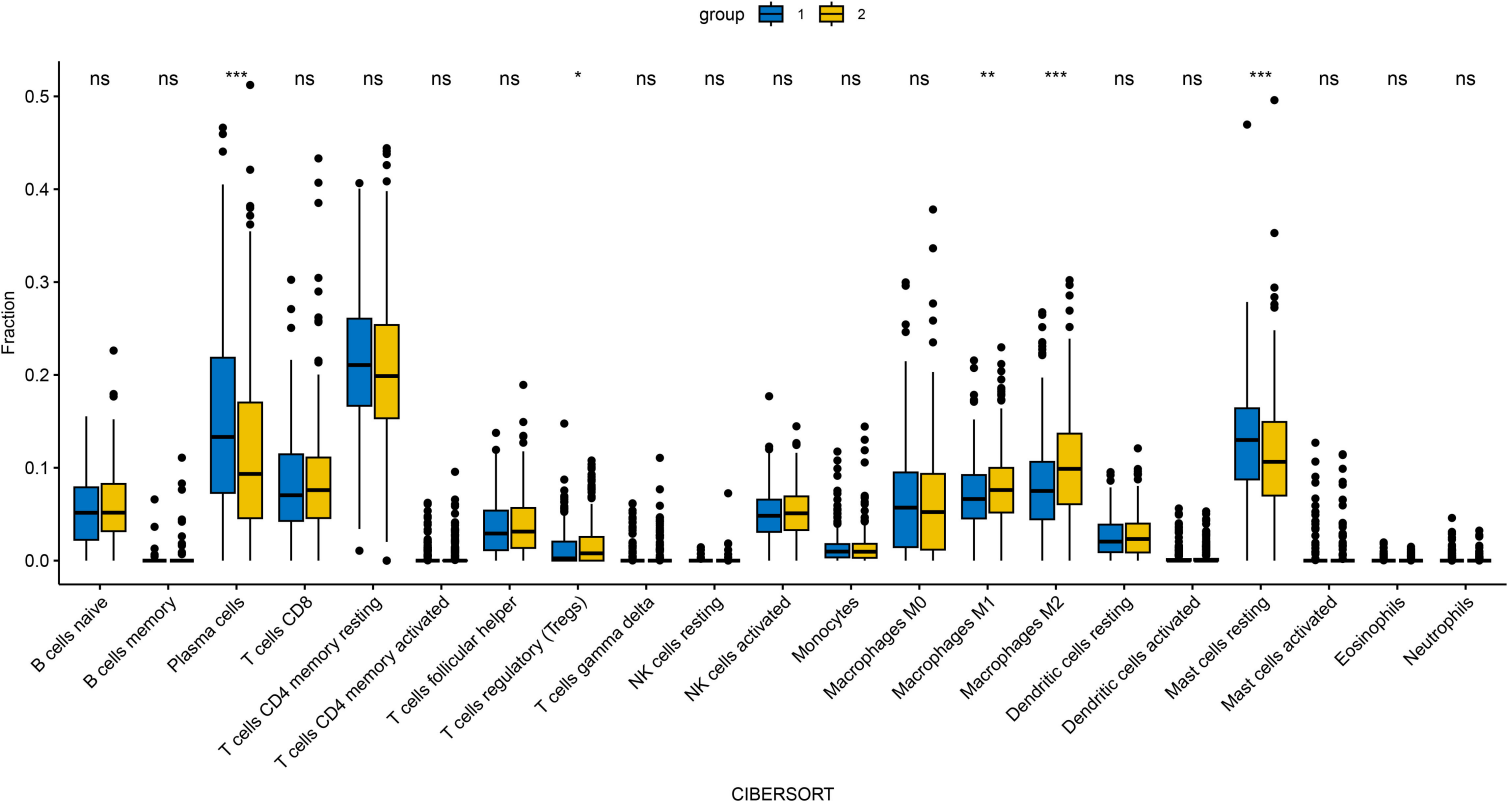
Immune cell infiltration profiles (via CIBERSORT) across distinct clusters defined by DOCK3 and CDKN3 co-expression patterns. *p < 0.05, **p < 0.01, ***p < 0.001.

To investigate the clinical relevance and heterogeneity of tumors, we stratified samples based on DOCK3 and CDKN3 expression. High DOCK3 expression showed a significant positive correlation with tumor samples (compared to normal tissue, **Figure 8**) and metastatic status (p < 0.01), suggesting a role in tumorigenesis and dissemination. In contrast, high CDKN3 expression was significantly associated with higher T stage, N stage, and Gleason score (**Figure 9**, all p < 0.01), implicating it in local invasion and malignant progression. With the distribution of expression changes clearly illustrated through a heatmap (**Figure 10**, highlighting the distinct expression patterns of DOCK3, associated with metastatic promotion, and CDKN3, linked to local invasion), immune infiltration also varied significantly among clusters (**Figure 11, p** < 0.05).

#### External validation using GSE55945

**Figure 12:**
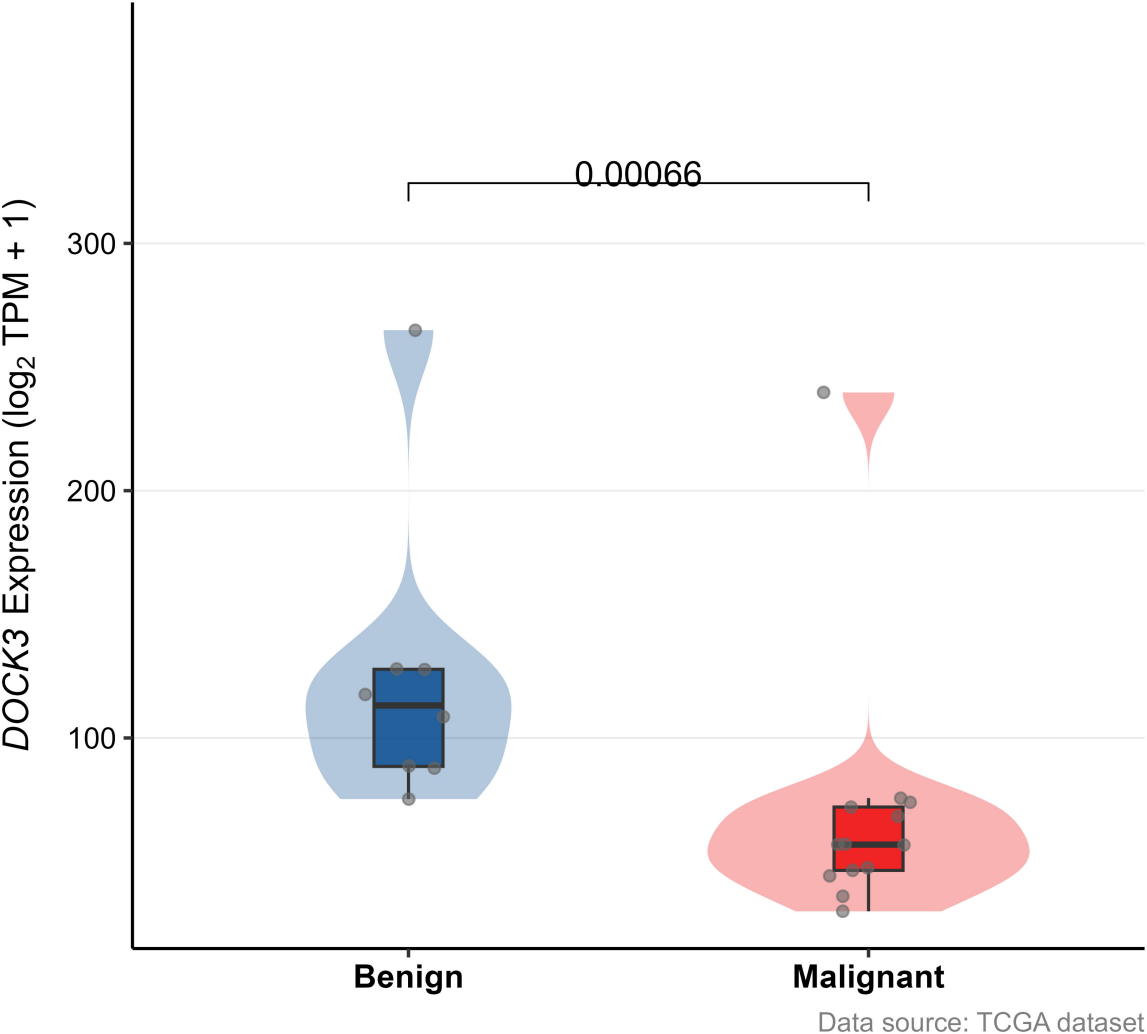
Validation of the association between gene expression (DOCK3) and clinical features in the external validation cohort (GSE55945).

To independently validate the expression pattern of DOCK3, we analyzed the GSE55945 dataset (**Figure 12**). DOCK3 expression was significantly elevated in prostate tumor compared to normal tissues (p < 0.05), supporting its potential role as a biomarker.

### Single-cell RNA-seq analysis results

**Figure 13:**
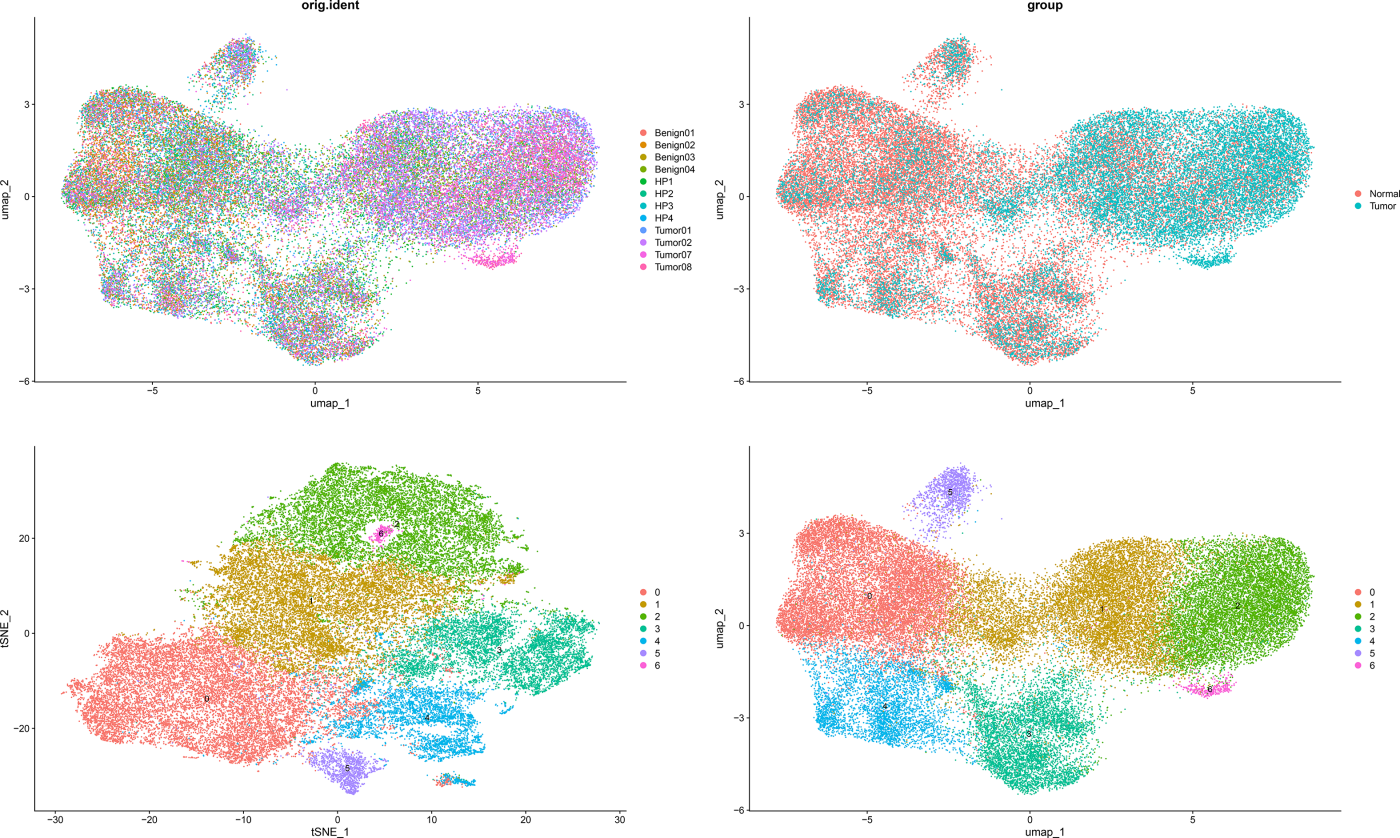
Dimensionality reduction plots (t-SNE and UMAP) showing single-cell clustering by sample of origin (orig.ident, left) and by biological condition (group, right).

To resolve cellular heterogeneity and identify cell type-specific expression of DOCK3, we performed single-cell RNA sequencing analysis. After quality control, 45,325 high-quality cells were retained. Unsupervised clustering identified 7 distinct cell clusters (**Figure 13**). Cluster 6 was specifically annotated as a malignant epithelial/stromal cluster derived from tumor samples. DOCK3 showed highly specific and significant overexpression in this cluster, with an average log2FC = 9.13 (adjusted p-value < 1e-200). The proportion of DOCK3-expressing cells in Cluster 6 (54%) was markedly higher than in all other clusters (< 0.1%).

## Conclusion

This study conducted a multi-dimensional investigation of the DOCK3 gene in PCa utilizing both bulk and single-cell transcriptomic analyses. At the bulk RNA level, DOCK3 was associated with distant metastasis and immune infiltration. Genes within its co-expression module (green module) were significantly enriched in pathways related to muscle contraction, cytoskeleton organization, and metabolic processes. High DOCK3 expression correlated with an activated tumor immune microenvironment, suggesting its potential as a therapeutic target for PCa immunotherapy. Single-cell analysis further validated the highly specific overexpression of DOCK3 within the malignant epithelial/stromal cell cluster (Cluster 6), providing robust cellular-level evidence for DOCK3 as a hallmark gene in PCa. These findings offer important theoretical insights into the mechanisms underlying PCa progression and provide a potential biomarker for exploring precision therapeutic strategies.

## Discussion

PCa exhibits low immunogenicity, with limited T-cell infiltration and an immunosuppressive tumor microenvironment, which partly explains the modest efficacy of immune checkpoint inhibitors (12, 13). Emerging evidence suggested that immune evasion mechanisms—such as PD-L1 upregulation and TGF-β signaling—facilitate tumor progression (14). In this context, DOCK3 may represent a novel modulator, potentially contributing to both metastatic progression and immune escape by altering immune regulatory pathways, thereby offering a new target for combinatorial immunotherapy (15).

The DOCK family, particularly the DOCK-B subfamily comprising DOCK3 and DOCK4, plays a key role in activating small GTPases such as Rac1 and Cdc42, which are crucial for regulating cytoskeletal dynamics and immune cell functions (16, 17). Research demonstrated that DOCK3, which is typically expressed in neural tissues, also plays a significant role in immune cells like T cells and macrophages, impacting their ability to migrate and respond to tumor cells (18). Such immune surveillance is pivotal in controlling tumor progression and in modulating responses to immunotherapies such as checkpoint inhibitors. Tumor cells frequently exploit multiple strategies to escape immune surveillance (19). By regulating cell movement and actin dynamics, DOCK3 can facilitate tumor cell invasion and metastasis. Studies have shown that DOCK3 overexpression enhances the aggressiveness of tumors, including melanomas, by promoting amoeboid invasion (5). Moreover, the activation of Rac and Cdc42 by DOCK proteins not only influences tumor cell motility but also reshapes the immune microenvironment by attracting immunosuppressive cells, thereby promoting immune evasion (20, 21).

Our study identified DOCK3 as a crucial player in PCa, particularly highlighting its ​novel dual role in promoting metastasis while concurrently modulating immune infiltration—a phenomenon not extensively reported in prostate cancer prior to this work. By integrating bulk RNA-sequencing (TCGA-PRAD), single-cell RNA-sequencing (GEO), and genomic data, we explored the expression characteristics of DOCK3 in prostate cancer and its potential as a therapeutic target. At the single-cell level, DOCK3 was specifically enriched in malignant epithelial cluster 6, which was largely composed of tumor-derived cells and lacked expression of benign epithelial markers. Co-expression with known tumor markers (e.g., KLK3, MMP9) reinforces DOCK3’s tumor-specific expression signature. This overexpression correlates with enhanced tumor cell migration, invasion, and cytoskeletal reorganization, which is consistent with the conclusions drawn from non-small cell lung cancer. DOCK3 was shown to promote invasion via RAC1 and is directly regulated by tumor-suppressive miRNAs such as miR-512-3p (11).

Clinical analysis further demonstrated that DOCK3 co-expression with CDKN3 defines a PCa subgroup enriched for high Gleason scores (≥8) and advanced T stage (T3/T4), underscoring its prognostic potential. These findings align with previous reports implicating DOCK3 in tumor cell migration and β-catenin–WAVE2 signaling in other cancers (22). Importantly, DOCK3 overexpression was associated with enhanced immune infiltration. Three independent immune deconvolution algorithms (CIBERSORT, ssGSEA, and xCell) consistently demonstrated that DOCK3-high tumors showed increased infiltration by cytotoxic CD8+ T cells and NK cells. This paradoxical observation—where a gene associated with metastasis also correlates with immune activation—raises intriguing questions. It is plausible that DOCK3-driven structural changes in the tumor architecture enhance immune cell recruitment, or alternatively, DOCK3 may be involved in immune-editing processes that support immune evasion despite apparent infiltration. Collectively, these findings indicated that DOCK3 functions not as a passive bystander but as a pivotal integrator of tumor progression signals and immune microenvironment remodeling.

These insights position DOCK3 as both a promising therapeutic target and a clinically actionable biomarker for patient stratification, especially in high-risk PCa where conventional immunotherapy often shows limited efficacy. Targeting DOCK3 may provide a synergistic approach to enhance immune checkpoint responsiveness and mitigate metastatic progression in immunologically “cold” tumors (23).

Several limitations should be acknowledged. Firstly, this study is based on retrospective analyses of publicly available transcriptomic and clinical datasets; thus, causal relationships cannot be firmly established. Nevertheless, the consistency of our findings across multiple independent algorithms and cohorts supports the robustness of the conclusions. Secondly, although we observed consistent associations between DOCK3 expression and immune infiltration, direct mechanistic insights into how DOCK3 modulates immune cell behavior are lacking. This gap, however, opens clear avenues for future experimental validation using CRISPR-based editing, immune co-culture models, or *in vivo* systems. Thirdly, functional assays were not conducted in this study, but the strong clinical and multi-omics correlations provide a solid foundation for such follow-up work. Additionally, tools like cellphoneDB could be employed in the future to explore DOCK3-mediated cell-cell communication networks, offering deeper insights into its role in immune modulation and tumor progression. These limitations do not diminish the validity of our results but instead highlight translational opportunities for functional studies and therapeutic exploration.

In conclusion, our comprehensive multi-omics analysis reveals DOCK3 as a critical nexus connecting genomic instability, metastatic behavior, and immune microenvironment remodeling in prostate cancer. Elevated DOCK3 expression correlates with both tumor aggressiveness and increased infiltration of cytotoxic immune cells, suggesting a dual role in promoting tumor progression while shaping the immune landscape. These findings underscored the potential of DOCK3 as a prognostic biomarker and a candidate immunotherapy target, particularly for stratifying patients who may benefit from immune-based treatments.

## Data Availability

The datasets presented in this study can be found in online repositories. The names of the repository/repositories and accession number(s) can be found below: https://www.ncbi.nlm.nih.gov/, GSE181294.
